# Metabolomic insights into pulmonary fibrosis: a mendelian randomization study

**DOI:** 10.1186/s12890-024-03079-6

**Published:** 2024-06-06

**Authors:** Wuyinuo Tang, Huanyu Jiang, Xinhui Wu, Guanyi Wu, Chenchong Zhao, Wenbo Lin, Ying Zhu, Guowei Jiang, Xiuhua Chen, Hang Huang, Lvyuan He

**Affiliations:** 1 Department of Pulmonology, Jinhua TCM Hospital Affiliated to Zhejiang Chinese Medical University, Jinhua, Zhejiang China; 2 School of basic medicine, Chengdu University of Traditional Chinese Medicine, Chengdu, Sichuan China; 3 Department of Geriatric, Hospital of Chengdu University of Traditional Chinese Medicine, Chengdu, Sichuan China

**Keywords:** Pulmonary fibrosis, Mendelian randomization, Metabolites, Genetic variants, Causal inference, GWAS, Statistical analysis

## Abstract

**Background:**

This study leverages a two-sample Mendelian Randomization (MR) approach to explore the causal relationships between 1,400 metabolites and pulmonary fibrosis, using genetic variation as instrumental variables. By adhering to stringent criteria for instrumental variable selection, the research aims to uncover metabolic pathways that may influence the risk and progression of pulmonary fibrosis, providing insights into potential therapeutic targets.

**Methods:**

Utilizing data from the OpenGWAS project, which includes a significant European cohort, and metabolite GWAS data from the Canadian Longitudinal Aging Study (CLSA), the study employs advanced statistical methods. These include inverse variance weighting (IVW), weighted median estimations, and comprehensive sensitivity analyses conducted using the R software environment to ensure the robustness of the causal inferences.

**Results:**

The study identified 62 metabolites with significant causal relationships with pulmonary fibrosis, highlighting both risk-enhancing and protective metabolic factors. This extensive list of metabolites presents a broad spectrum of potential therapeutic targets and biomarkers for early detection, underscoring the metabolic complexity underlying pulmonary fibrosis.

**Conclusions:**

The findings from this MR study significantly advance our understanding of the metabolic underpinnings of pulmonary fibrosis, suggesting that alterations in specific metabolites could influence the risk and progression of the disease. These insights pave the way for the development of novel diagnostic and therapeutic strategies, emphasizing the potential of metabolic modulation in managing pulmonary fibrosis.

**Supplementary Information:**

The online version contains supplementary material available at 10.1186/s12890-024-03079-6.

## Introduction

Pulmonary fibrosis represents a major global health burden, with rising prevalence and high associated morbidity and mortality. Idiopathic pulmonary fibrosis (IPF), the most common subtype, affects over 3 million individuals worldwide [1]. Hallmarked by progressive scarring and stiffening of the lungs, pulmonary fibrosis leads to impaired gas exchange, respiratory failure, and eventual death within 3–5 years of diagnosis for IPF patients [2]. The limited treatment options and poor prognosis underscore the need for greater insight into IPF pathogenesis to enable early detection and inform novel therapeutic targets.

Emerging evidence implicates metabolic dysregulation as a potential contributor to pulmonary fibrotic processes [3]. Metabolomic profiling studies reveal perturbations in multiple metabolic pathways in IPF patients versus controls, including alterations in glycolysis, lipogenesis, proteolysis, and redox balance [4, 5]. Deranged cellular metabolism may disrupt homeostatic pathways in epithelial cells, fibroblasts, and immune cells within the lung, influencing mechanisms linked to fibrosis like inflammation, apoptosis, proliferation, and collagen deposition [6, 7]. Elucidating the specific metabolic pathways involved could shed light on IPF development while identifying candidate biomarkers and therapeutic opportunities.

However, observational associations between metabolites and pulmonary fibrosis are susceptible to confounding and reverse causation, which obscure causal relationships [8]. MR analysis leverages genetic variation to strengthen causal inference regarding exposure-outcome associations in observational data [9]. Single nucleotide polymorphisms (SNPs) linked to specific metabolites can serve as instrumental variables (IVs) to model the influence of genetically-determined exposures on disease outcomes. Since genotypes are randomly allocated before disease onset, MR studies minimize biases from confounding and reverse causation [10].

This study performs a comprehensive MR analysis exploring potential causal relationships between 1,400 blood metabolites and pulmonary fibrosis risk. It combines metabolite genome-wide association study (GWAS) data from over 8,000 participants in the Canadian Longitudinal Study on Aging (CLSA) with extensive genetic and pulmonary fibrosis data from the expansive OpenGWAS database [11, 12]. The analysis scans across a broad panel of metabolites to provide an agnostic overview of metabolic pathways that may be etiologically involved in pulmonary fibrosis pathogenesis. The use of two-sample MR retains statistical power by extracting summary data for metabolites and disease outcomes from separate large-scale cohorts.

The study implements a rigorous framework for genetic IV selection, sensitivity analyses, and causal modeling to ensure robust causal associations. Findings could identify promising circulating biomarkers for early pulmonary fibrosis detection along with potential preventive targets. By delineating specific metabolites with causal implications for fibrosis, the study promises to uncover key metabolic derangements driving lung injury and progression in IPF. These insights may pave the way for novel interventions that target pathogenic metabolic pathways to halt or reverse the progression of this devastating disease.

## Materials and methods

### Study design

In a detailed two-sample MR study, researchers scrutinized the causal connections between 1,400 metabolites and pulmonary fibrosis, utilizing genetic variations as proxies for these metabolites. To ensure the integrity of the analysis, the study adhered to three critical assumptions essential for valid causal inference: genetic variations must be closely linked to the metabolites in question, not related to confounders that could skew the relationship between these metabolites and pulmonary fibrosis, and influence the disease exclusively through these metabolites [9, 12].

Leveraging data from the OpenGWAS database (https://gwas.mrcieu.ac.uk/datasets/ebi-a-GCST90018908/), which encompasses 469,126 Europeans, including 1,566 individuals diagnosed with pulmonary fibrosis and 467,560 controls, this study aimed to unearth genetic markers that could shed light on the metabolic underpinnings of pulmonary fibrosis. This large-scale analysis not only enhances our understanding of the disease’s genetic and metabolic framework but also sets the stage for identifying potential biomarkers and therapeutic targets for pulmonary fibrosis. The data for IPF and lung function are also from IEU openGWAS. The ID numbers for the two groups of idiopathic pulmonary fibrosis are ebi-a-GCST90018120 and finn-b-IPF, respectively, and the ID number for lung function is ebi-a-GCST90029026.

### Metabolite GWAS data sources

The GWAS catalog (https://www.ebi.ac.uk/gwas/), providing aggregate statistics for metabolites with registration numbers from GCST90199621 to GCST90201020, showcases a comprehensive GWAS study of 1,091 metabolites and 309 metabolite ratios from 8,299 individuals in the Canadian Longitudinal Aging Study (CLSA) cohort. This large-scale study is pivotal for understanding the genetic determinants of metabolic traits in a representative sample of the population, facilitating insights into metabolic regulation, disease risk biomarkers, and potential therapeutic targets [13]. The inclusion of a wide array of metabolites and ratios, analyzed in a well-characterized cohort, enhances the depth and applicability of the findings, providing a nuanced view of metabolic processes.

### Selection of instrumental variables (IVs)

Since genetic variation is directly related to exposure, the significance level of IVs for each metabolite was set at 1 × 10^− 5^, the significance level of IVs for each metabolite was set at 1 × 10^− 5^. To obtain IVs for independent sites, we used the “TwoSampleMR” packet data with a linkage unbalance (LD) threshold set to R^2^ < 0.001 and an aggregation distance of 10,000 kb [10]. For Pulmonary fibrosis, we adjusted the significance level to 5 × 10^− 6^, which is commonly used to represent genome-wide significance in GWAS, with a LD threshold of R^2^ < 0.001 and an aggregation distance of 10,000 kb [14]. For IPF, ebi-a-GCST90018120, we use the screening criteria of 5 × 10^− 6^, for finn-b-IPF, we use the screening criteria of 5 × 10^− 8^, and for lung function, we also use the screening criteria of 5 × 10^− 8^.

### Statistical analysis

**Fig. 1:**
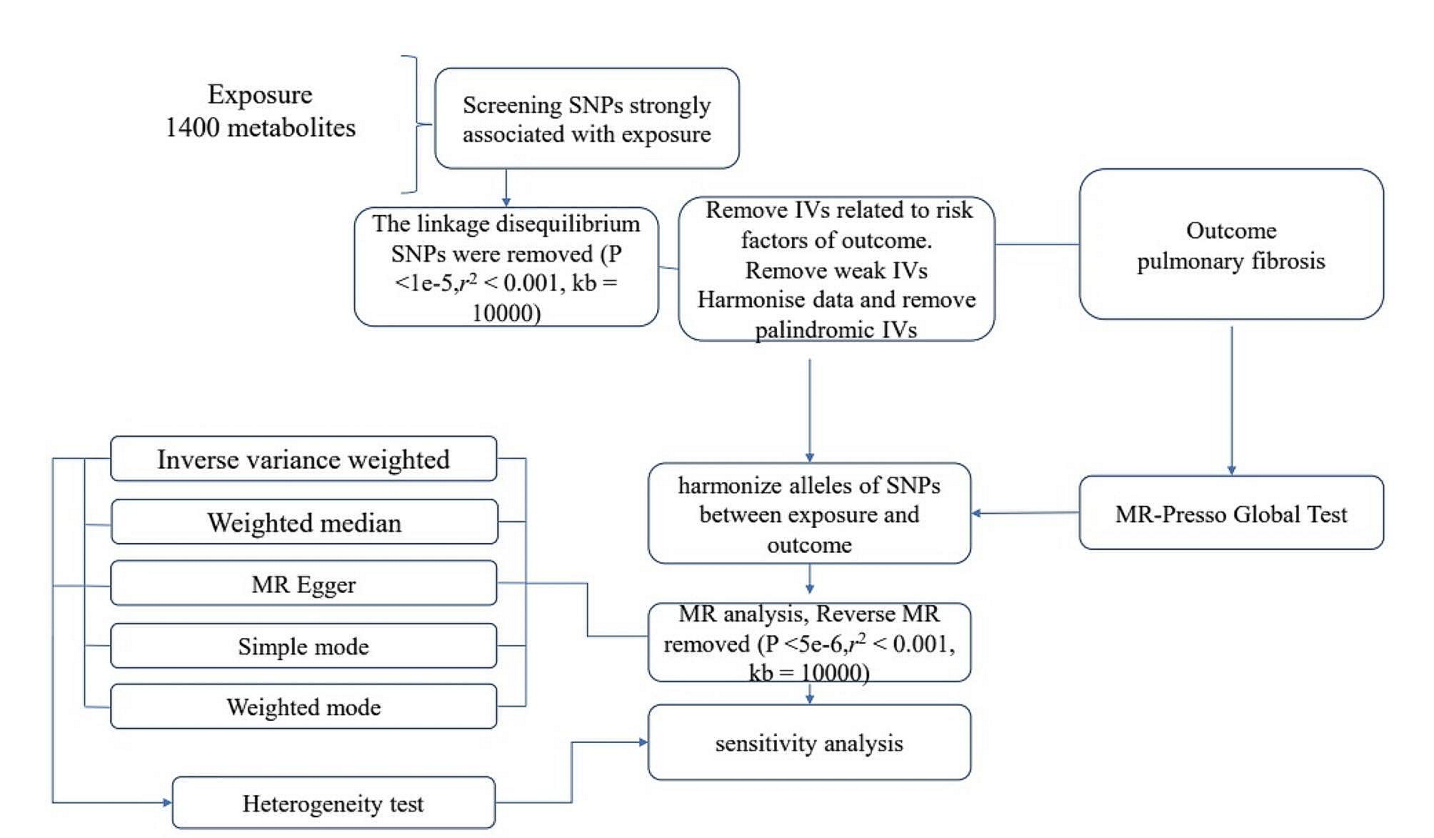
Flow diagram for quality control of the instrumental variables (IVs) and the entire Mendelian Randomization (MR) analysis process. *Abbreviations: SNPs, single-nucleotide polymorphisms; IVW, inverse variance weighted; MR, Mendelian Randomization; MR Presso, Mendelian Randomization Pleiotropy RESidual Sum and Outlier

For the statistical analysis portion of our study examining the causal influence of metabolite on Pulmonary fibrosis risk, all procedures were conducted using R software, version 4.2.1, which is a widely used environment for statistical computing and graphics, available at (http://www.Rproject.org) [15]. To ascertain the causal relationships between the 1400 metabolite and Pulmonary fibrosis, we primarily employed methods including inverse variance weighting (IVW), weighted median-based estimation. These analyses were facilitated by the ' TwoSampleMR ' package, version 0.5.7, within the R software environment [16]. This package is specifically designed for conducting MR analyses, providing tools for estimation, testing, and sensitivity analysis of causal effects. The IVW method is a standard approach in MR that combines the Wald estimates (ratio of the SNP-outcome association to the SNP-exposure association) from multiple genetic variants, weighting by the inverse variance of each SNP-outcome association [17]. The weighted median and mode-based methods serve as supplementary approaches that provide robust causal estimates even when some of the instrumental variables are invalid, as long as certain assumptions are met. These analyses were backed up by rigorous sensitivity analyses, including Cochran’s Q test to examine heterogeneity amongst the instrumental variables [18]. Such thorough statistical evaluation ensures that the findings regarding the relationship between metabolite and Pulmonary fibrosis are as reliable and accurate as possible given the data. The whole process was shown in Fig. 1.

## Results

### Exploration of the causal effect of metabolite on pulmonary fibrosis risk

**Fig. 2:**
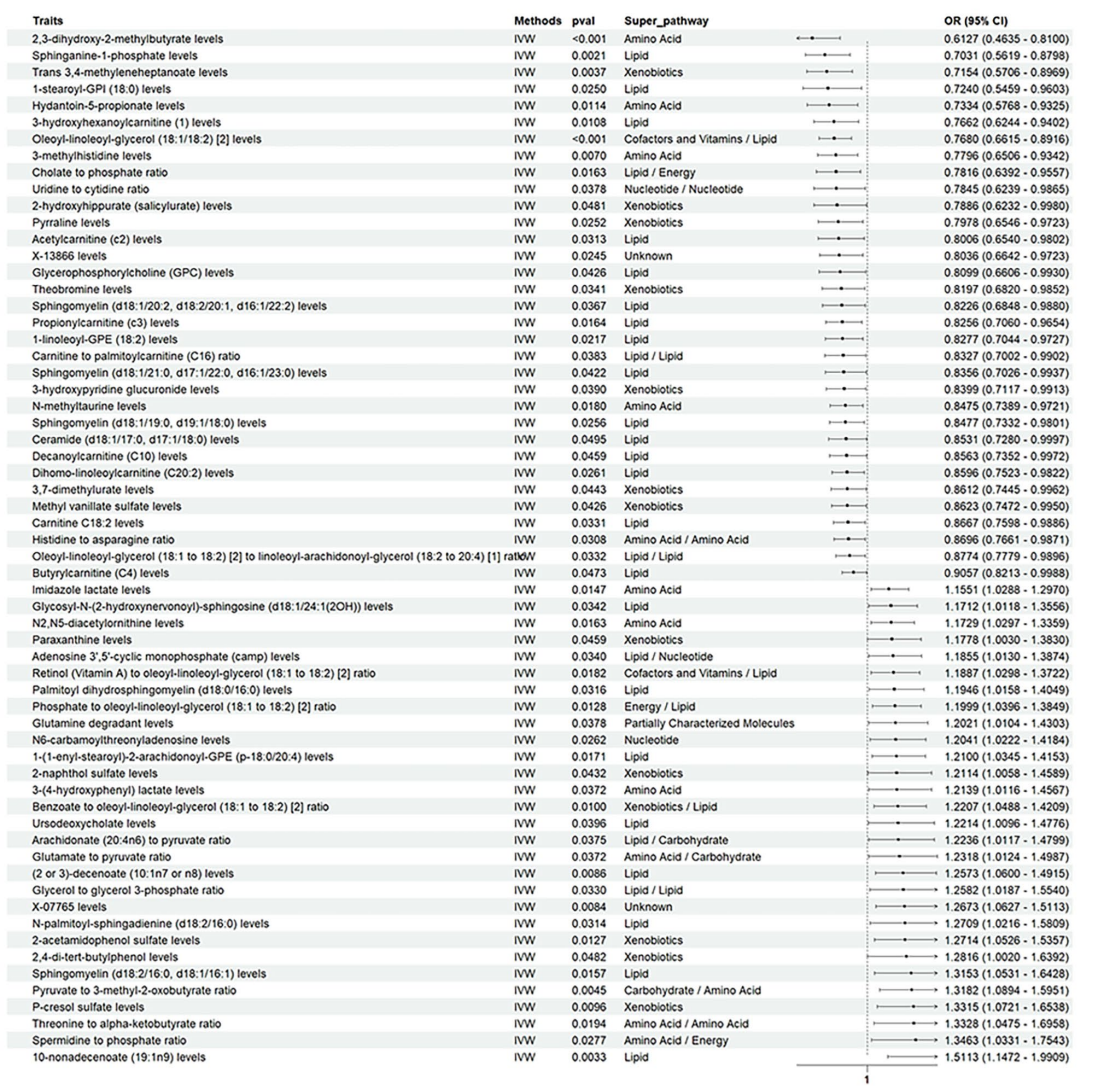
Forest plots depicting the causal associations between Pulmonary fibrosis and specific metabolite. *Abbreviations: IVW, inverse variance weighting; CI, confidence interval

At a predetermined significance level of 0.05, this study identified 62 metabolites that have a causal relationship with pulmonary fibrosis. This important discovery emphasizes the complexity of the metabolic basis of the disease and highlights potential targets for therapeutic interventions and biomarkers for early detection. We also classified positive results using Super_pathway. (as shown in Fig. 2).

The N2,N5-diacetylornithine levels (*P* = 0.016, OR = 1.172, 95%CI = 1.029 ~ 1.335), 2-acetamidophenol sulfate levels (*P* = 0.012, OR = 1.271, 95%CI = 1.052 ~ 1.535), Palmitoyl dihydrosphingomyelin (d18:0/16:0) levels (*P* = 0.031, OR = 1.194, 95%CI = 1.015 ~ 1.404), 1-(1-enyl-stearoyl)-2-arachidonoyl-GPE (p-18:0/20:4) levels (*P* = 0.017, OR = 1.210, 95%CI = 1.034 ~ 1.415), N-palmitoyl-sphingadienine (d18:2/16:0) levels (*P* = 0.031, OR = 1.270, 95%CI = 1.021 ~ 1.580), Glycosyl-N-(2-hydroxynervonoyl)-sphingosine (d18:1/24:1(2OH)) levels (*P* = 0.034, OR = 1.171, 95%CI = 1.011 ~ 1.355), 2-naphthol sulfate levels (*P* = 0.043, OR = 1.211, 95%CI = 1.005 ~ 1.458), (2 or 3)-decenoate (10:1n7 or n8) levels (*P* = 0.008, OR = 1.257, 95%CI = 1.059 ~ 1.491), 2,4-di-tert-butylphenol levels (*P* = 0.048, OR = 1.281, 95%CI = 1.001 ~ 1.639), Ursodeoxycholate levels (*P* = 0.039, OR = 1.221, 95%CI = 1.009 ~ 1.477), Adenosine 3’,5’-cyclic monophosphate (camp) levels (*P* = 0.033, OR = 1.185, 95%CI = 1.012 ~ 1.387), 3-(4-hydroxyphenyl)lactate levels (*P* = 0.037, OR = 1.213, 95%CI = 1.011 ~ 1.456), X-07765 levels (*P* = 0.008, OR = 1.267, 95%CI = 1.062 ~ 1.511), Arachidonate (20:4n6) to pyruvate ratio (*P* = 0.037, OR = 1.223, 95%CI = 1.011 ~ 1.479), Pyruvate to 3-methyl-2-oxobutyrate ratio (*P* = 0.004, OR = 1.318, 95%CI = 1.089 ~ 1.595), Spermidine to phosphate ratio (*P* = 0.027, OR = 1.346, 95%CI = 1.033 ~ 1.754), Glycerol to glycerol 3-phosphate ratio (*P* = 0.033, OR = 1.258, 95%CI = 1.018 ~ 1.554), Phosphate to oleoyl-linoleoyl-glycerol (18:1 to 18:2) [2] ratio (*P* = 0.012, OR = 1.199, 95%CI = 1.039 ~ 1.384), Retinol (Vitamin A) to oleoyl-linoleoyl-glycerol (18:1 to 18:2) [2] ratio (*P* = 0.018, OR = 1.188, 95%CI = 1.029 ~ 1.372), Glutamate to pyruvate ratio (*P* = 0.037, OR = 1.231, 95%CI = 1.012 ~ 1.498), Benzoate to oleoyl-linoleoyl-glycerol (18:1 to 18:2) [2] ratio (*P* = 0.010, OR = 1.220, 95%CI = 1.048 ~ 1.420), Threonine to alpha-ketobutyrate ratio (*P* = 0.019, OR = 1.332, 95%CI = 1.047 ~ 1.695), Imidazole lactate levels (*P* = 0.014, OR = 1.155, 95%CI = 1.028 ~ 1.296), Paraxanthine levels in elite athletes (*P* = 0.045, OR = 1.177, 95%CI = 1.002 ~ 1.383), 10-nonadecenoate (19:1n9) levels (*P* = 0.003, OR = 1.511, 95%CI = 1.147 ~ 1.990), P-cresol sulfate levels (*P* = 0.009, OR = 1.331, 95%CI = 1.072 ~ 1.653), N6-carbamoylthreonyladenosine levels (*P* = 0.026, OR = 1.204, 95%CI = 1.022 ~ 1.418), Glutamine degradant levels (*P* = 0.037, OR = 1.202, 95%CI = 1.010 ~ 1.430), Sphingomyelin (d18:2/16:0, d18:1/16:1) levels (*P* = 0.015, OR = 1.315, 95%CI = 1.053 ~ 1.642) are positively correlated with Pulmonary fibrosis.

While Sphingomyelin (d18:1/20:2, d18:2/20:1, d16:1/22:2) levels (*P* = 0.036, OR = 0.822, 95%CI = 0.684 ~ 0.987), Trans 3,4-methyleneheptanoate levels (*P* = 0.003, OR = 0.715, 95%CI = 0.570 ~ 0.896), Sphingomyelin (d18:1/21:0, d17:1/22:0, d16:1/23:0) levels (*P* = 0.042, OR = 0.835, 95%CI = 0.702 ~ 0.993),2,3-dihydroxy-2-methylbutyrate levels (*P* = 0.000, OR = 0.612, 95%CI = 0.463 ~ 0.809), Ceramide (d18:1/17:0, d17:1/18:0) levels (*P* = 0.049, OR = 0.853, 95%CI = 0.728 ~ 0.999), Sphingomyelin (d18:1/19:0, d19:1/18:0) levels (*P* = 0.025, OR = 0.847, 95%CI = 0.733 ~ 0.980), Dihomo-linoleoylcarnitine (C20:2) levels (*P* = 0.026, OR = 0.859, 95%CI = 0.752 ~ 0.982), Methyl vanillate sulfate levels (*P* = 0.042, OR = 0.862, 95%CI = 0.747 ~ 0.995), 3-hydroxypyridine glucuronide levels (*P* = 0.039, OR = 0.839, 95%CI = 0.711 ~ 0.991), 3-hydroxyhexanoylcarnitine [1] levels (*P* = 0.010, OR = 0.766, 95%CI = 0.624 ~ 0.940), 2-hydroxyhippurate (salicylurate) levels (*P* = 0.048, OR = 0.788, 95%CI = 0.623 ~ 0.997), X-13,866 levels (*P* = 0.024, OR = 0.803, 95%CI = 0.664 ~ 0.972), Carnitine C4 levels (*P* = 0.047, OR = 0.905, 95%CI = 0.821 ~ 0.998), Uridine to cytidine ratio (*P* = 0.037,OR = 0.784, 95%CI = 0.623 ~ 0.986), Carnitine to palmitoylcarnitine (C16) ratio (*P* = 0.038, OR = 0.832, 95%CI = 0.700 ~ 0.990), Oleoyl-linoleoyl-glycerol (18:1 to 18:2) [2] to linoleoyl-arachidonoyl-glycerol (18:2 to 20:4) [1] ratio (*P* = 0.033, OR = 0.877, 95%CI = 0.777 ~ 0.989), Cholate to phosphate ratio (*P* = 0.016, OR = 0.781, 95%CI = 0.639 ~ 0.955), Histidine to asparagine ratio (*P* = 0.030, OR = 0.869, 95%CI = 0.766 ~ 0.987), 3-methylhistidine levels (*P* = 0.006, OR = 0.779, 95%CI = 0.650 ~ 0.934), Glycerophosphorylcholine (GPC) levels (*P* = 0.042, OR = 0.809, 95%CI = 0.660 ~ 0.993), Theobromine levels (*P* = 0.034, OR = 0.819, 95%CI = 0.681 ~ 0.985), 1-stearoyl-GPI (18:0) levels (*P* = 0.025, OR = 0.724, 95%CI = 0.545 ~ 0.960), Acetylcarnitine levels (Biocrates platform) (*P* = 0.031, OR = 0.800, 95%CI = 0.653 ~ 0.980), Propionylcarnitine (c3) levels (*P* = 0.016, OR = 0.825 95%, CI = 0.705 ~ 0.965), Decanoylcarnitine (C10) levels (*P* = 0.045, OR = 0.856, 95%CI = 0.735 ~ 0.997), 3,7-dimethylurate levels (*P* = 0.044, OR = 0.861, 95%CI = 0.744 ~ 0.996), 1-linoleoyl-GPE (18:2) levels (*P* = 0.021, OR = 0.827, 95%CI = 0.704 ~ 0.972), Pyrraline levels (*P* = 0.025, OR = 0.797, 95%CI = 0.654 ~ 0.972), Sphinganine-1-phosphate levels (*P* = 0.002,OR = 0.703, 95%CI = 0.561 ~ 0.879), Hydantoin-5-propionate levels (*P* = 0.011, OR = 0.733, 95%CI = 0.576 ~ 0.932), Oleoyl-linoleoyl-glycerol (18:1/18:2) [2] levels (*P* = 0.000, OR = 0.767, 95%CI = 0.661 ~ 0.891), Carnitine C18:2 levels (*P* = 0.033, OR = 0.866, 95%CI = 0.759 ~ 0.988), N-methyltaurine levels (*P* = 0.018, OR = 0.847, 95%CI = 0.738 ~ 0.972) are inversely associated with Pulmonary fibrosis. Results from sensitivity analyses demonstrate therobustness of the observed causalassociation (Supplementary Material. 1). Scatter plot, funnel plot and forest plot also show the stability of the results (Supplementary Material. 1).

### Exploration of the causal effect of pulmonary fibrosis risk on metabolite

**Fig. 3:**
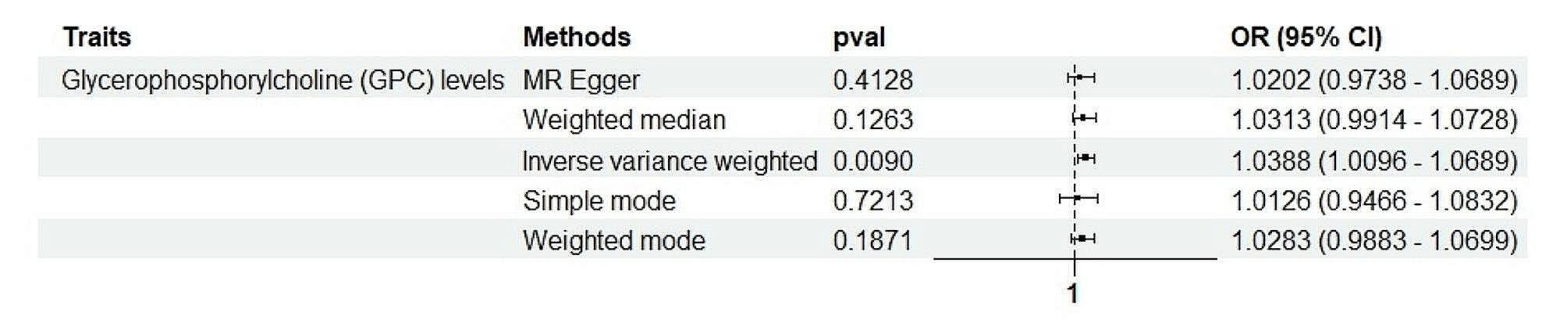
Forest plots depicting the causal associations between specific metabolite and Pulmonary fibrosis. *Abbreviations: IVW, inverse variance weighting; CI, confidence interval

To further deepen the understanding of these complex interactions, a reverse MR analysis was conducted. This approach aimed to discern whether the onset of pulmonary fibrosis could, in turn, influence the levels of the previously identified 62 metabolites that showed a causal relationship with the disease. Such an analysis is pivotal in distinguishing between metabolites that may contribute to disease pathogenesis and those that may be altered as a consequence of the disease process itself (as shown in Fig. 3).

Among the notable findings from this comprehensive analysis was the identification of a positive correlation between pulmonary fibrosis and Glycerophosphorylcholine (GPC) levels, as indicated by a *P*-value of 0.033, an Odds Ratio (OR) of 1.038, and a 95% Confidence Interval (CI) of 1.009 to 1.068. This result is particularly intriguing, as GPC is a metabolite involved in phospholipid metabolism and has been implicated in various biological processes, including cell membrane integrity and signaling pathways.

The association between increased GPC levels and pulmonary fibrosis suggests that alterations in phospholipid metabolism may play a role in the disease’s pathogenesis or progression. GPC’s involvement in maintaining cell membrane structure and function could influence the fibrotic processes characteristic of pulmonary fibrosis, potentially offering a novel biomarker for early detection or a target for therapeutic intervention.

### The causal relationship between metabolites and IPF and lung function

We conducted a two sample Mendelian randomization analysis using 1400 metabolites and two sets of IPF data, and then compared the results with the aforementioned PF. We found that the Benzoate to oleoyl linoleyl glycerol (18:1 to 18:2) [2] ratio could promote disease progression in both sets of IPF and PF results (Fig. 2, Supplementary Material 2, Supplementary Material 3). Afterwards, we explored the causal relationship between metabolites and Lung function (FEV_1_/FVC), and found that there were 91 metabolites that had a causal relationship with Lung function (FEV_1_/FVC). Among them, 42 metabolites had a negative causal relationship with Lung function (FEV_1_/FVC), while 49 metabolites had a positive causal relationship with Lung function (FEV_1_/FVC) (Supplementary Material 4).

### Reliability evaluation results

In this comprehensive analysis, the application of the Inverse Variance Weighting (IVW) method within a random effects model allowed for the consideration of heterogeneity across instrumental variables (IVs) without compromising the integrity of the causal inferences made between metabolites and pulmonary fibrosis. This strategic choice underscores the robustness of the analytical framework, effectively accommodating the variability inherent in genetic data.

To further validate the reliability of the causal relationships identified, the study employed the MR-Egger intercept test, a critical tool for detecting the presence of horizontal pleiotropy—a condition where genetic variants affect the outcome through pathways other than the exposure of interest. The findings, indicating no significant horizontal pleiotropy (*P* > 0.05), bolster the argument that the genetic variations selected as IVs genuinely represent the influence of the metabolites on pulmonary fibrosis, free from confounding biases.

The rigorousness of the analysis was further enhanced by conducting a sensitivity analysis using the leave-one-out method. This approach, essential for assessing the impact of individual IVs on the overall results, revealed no outliers, affirming the stability and consistency of the causal links established between the metabolites and pulmonary fibrosis.

## Discussion

The current study provides compelling evidence for a causal role of metabolic dysregulation in the pathogenesis and progression of pulmonary fibrosis. By implementing a rigorous two-sample Mendelian randomization approach, the analysis identified 62 metabolites exhibiting significant causal associations with pulmonary fibrosis risk. These findings deliver key insights into the complex metabolic underpinnings of this devastating lung disease, highlighting promising therapeutic targets and biomarker candidates.

A foremost implication of the results is the confirmation of extensive metabolic perturbations as likely contributors to fibrotic processes in the lung. The wide array of metabolites implicated spans multiple pathways, including amino acid, lipid, carbohydrate, nucleotide, and redox metabolism. This reinforces an emerging paradigm shift acknowledging the influence of systemic and cellular metabolism on pulmonary fibrosis onset and advancement [6, 19]. The study’s agnostic approach facilitates an unbiased overview of metabolic pathways that may be causally involved, without limiting the scope of investigation.

In particular, the findings showcase amino acid metabolic dysregulation as a centerpiece of pulmonary fibrosis pathogenesis. The identification of causal relationships for metabolites of arginine (N2,N5-diacetylornithine), tryptophan (3-hydroxyanthranilate), phenylalanine (3-(4-hydroxyphenyl)lactate), and branched-chain amino acids (2-hydroxyisovalerate, 2-methylbutyrylcarnitine) aligns with existing evidence. Experimental studies demonstrate that amino acid catabolism gone awry can disrupt collagen production, immune cell function, and redox balance to instigate fibrotic injury [7, 20]. Our results provide a key validation of these mechanistic links in human disease, solidifying amino acid metabolism as a high-value target.

Intriguingly, the study reveals a causal role for nucleotide metabolites like N6-carbamoylthreonyladenosine and uridine in modulating fibrosis risk. This novel finding adds to emerging data on purinergic signaling in pulmonary fibrosis [21]. It suggests that derangements in pyrimidine or purine metabolism may influence immune cell activation, fibroblast proliferation, and epithelial cell survival to impact disease outcomes [22]. Further research should explore if strategies targeting nucleotide metabolic pathways could offer therapeutic benefit.

The identification of multiple lipid species, including sphingomyelins, ceramides, and glycerophospholipids, also aligns with accumulating evidence. Sphingolipid metabolites can regulate key pathways linked to lung fibrosis like inflammation, apoptosis, and myofibroblast differentiation [23]. Our study provides imperative causal validation in humans that lipid metabolic reprogramming plays a instigating role. Targeting sphingolipid metabolism could hence represent a disease-modifying strategy.

A key asset of the study is the execution of a reverse MR analysis, evaluating the effect of pulmonary fibrosis onset on metabolite levels. This discerns metabolites involved in disease development versus those altered as a consequence of pathology. Intriguingly, glycerophosphocholine emerged with a significant causal association from fibrosis to metabolite. This indicates that perturbations in phospholipid metabolism may occur secondary to the initiation of fibrotic processes. As such, glycerophosphocholine merits exploration as a biomarker for early disease detection or progression monitoring, rather than a causal contributor.

After conducting a two-sample Mendelian randomization analysis of 1400 metabolites with IPF and lung function, we found that the ratio of benzoate to oleoyl-linoleoyl-glycerol (18:1 to 18:2) [2] may promote the progression of pulmonary fibrosis. This result was significant in both sets of IPF data and the previously mentioned PF results, making it one of the most reliable findings of our analysis. Additionally, we explored the causal relationships between metabolites and lung function (FEV_1_/FVC ratio), finding that 91 metabolites had a causal relationship with lung function. Of these, 42 metabolites negatively impacted lung function, while 49 had a positive effect. These findings emphasize the potential significant role of metabolic regulation in the pathogenesis of pulmonary fibrosis. The increase in the benzoate to oleoyl-linoleoyl-glycerol ratio not only showed a consistent disease-promoting effect across two IPF datasets but also played a critical role in the overall analysis of pulmonary fibrosis risk. This suggests that intervening in metabolic pathways may offer new strategies for treating or mitigating this fatal disease. Therefore, further investigation into the specific role of this metabolic marker in disease mechanisms will be an important direction for future research.Overall, the study’s comprehensive mapping of metabolic pathways altered in pulmonary fibrosis enhances mechanistic understanding and highlights new therapeutic possibilities. A foremost direction is intervening upon metabolic processes showing causal effects on disease risk. For instance, modulating amino acid or sphingolipid metabolism through pharmacological or nutritional approaches may ameliorate fibrosis by restoring metabolic homeostasis [24]. A second key direction is utilizing causally implicated metabolites as biomarkers for early diagnosis, risk stratification, or therapeutic monitoring in IPF patients. Lastly, expanding analyses to diseased lung tissue or fibrotic lung cell models could offer further validation of key metabolic pathways.

A leading strength that enhances the reliability of the causal inferences is the use of genetic variants as instrumental variables. By leveraging Mendel’s laws of inheritance, MR studies overcome limitations of conventional observational research and strengthen casual conclusions [25]. Our analysis implements stringent criteria for IV selection, exclusion of pleiotropy, and application of robust statistical models. This circumvents biases from confounding, reverse causation, and chance associations that frequently distort observational findings.

The two-sample MR design further augments statistical power by utilizing summary data from large GWAS datasets for metabolite exposures and disease outcomes [26]. By focusing specifically on European cohorts, the study ensures ethnic homogeneity and minimizes confounding by population stratification. Moreover, the use of data from a population-based cohort like CLSA for metabolite GWAS enhances external validity of the findings. Altogether, the study design and analysis pipeline reinforce the reliability of the identified causal associations.

However, some limitations should be acknowledged. The main constraint is the lack of replication cohorts with extensive metabolomics data, which could help validate findings. While reverse causation bias was addressed, the influence of unmeasured confounding cannot be fully excluded. Additionally, exploring cell-type specific expression patterns of the prioritized metabolites could have offered deeper insight. *Lastly, the study examined overall pulmonary fibrosis risk, limiting conclusions about metabolites driving IPF specifically.* The strength of the association between the metabolites in this study and the risk of pulmonary fibrosis needs to be further explored by in vitro experiments or pathway analyses to validate the significance of these findings. Future analyses in IPF cohorts are warranted, though challenges obtaining large sample sizes exist.

In conclusion, the current two-sample MR study substantiates a causal role for metabolic dysregulation in pulmonary fibrosis development and progression. After comprehensive analysis, the final results found that benzoate to oleoyl-linoleoyl-glycerol (18:1 to 18:2) [2] ratio may promote the progression of pulmonary fibrosis. In addition, some metabolite and ratio levels are also considered as potential therapeutic targets for pulmonary fibrosis. It elucidates metabolic pathways that may instigate and propagate fibrotic injury. These findings illuminate novel targets for developing urgently needed prognostic biomarkers and disease-modifying therapies for this devastating illness. With further validation, interventions aimed at restoring metabolic homeostasis in the lung could pave the way for long-awaited breakthroughs in pulmonary fibrosis management.

### Electronic supplementary material

Below is the link to the electronic supplementary material.

Supplementary Material 1

Supplementary Material 2

Supplementary Material 3

Supplementary Material 4

## Data Availability

The original contributions presented in the study are included in the article/ Supplementary Material. Further inquiries can be directed to the corresponding author.
