# Supplementary material for: Metabolomic insights into pulmonary fibrosis: a mendelian randomization study
Source: BMC Pulm Med. 2024 Jun 6;24:271. doi: 10.1186/s12890-024-03079-6 (PMC11155030; doi:10.1186/s12890-024-03079-6)
Supplement: Supplementary file 1 — Supplementary Material 1 [file 12890_2024_3079_MOESM1_ESM.docx]

Supplementary Material


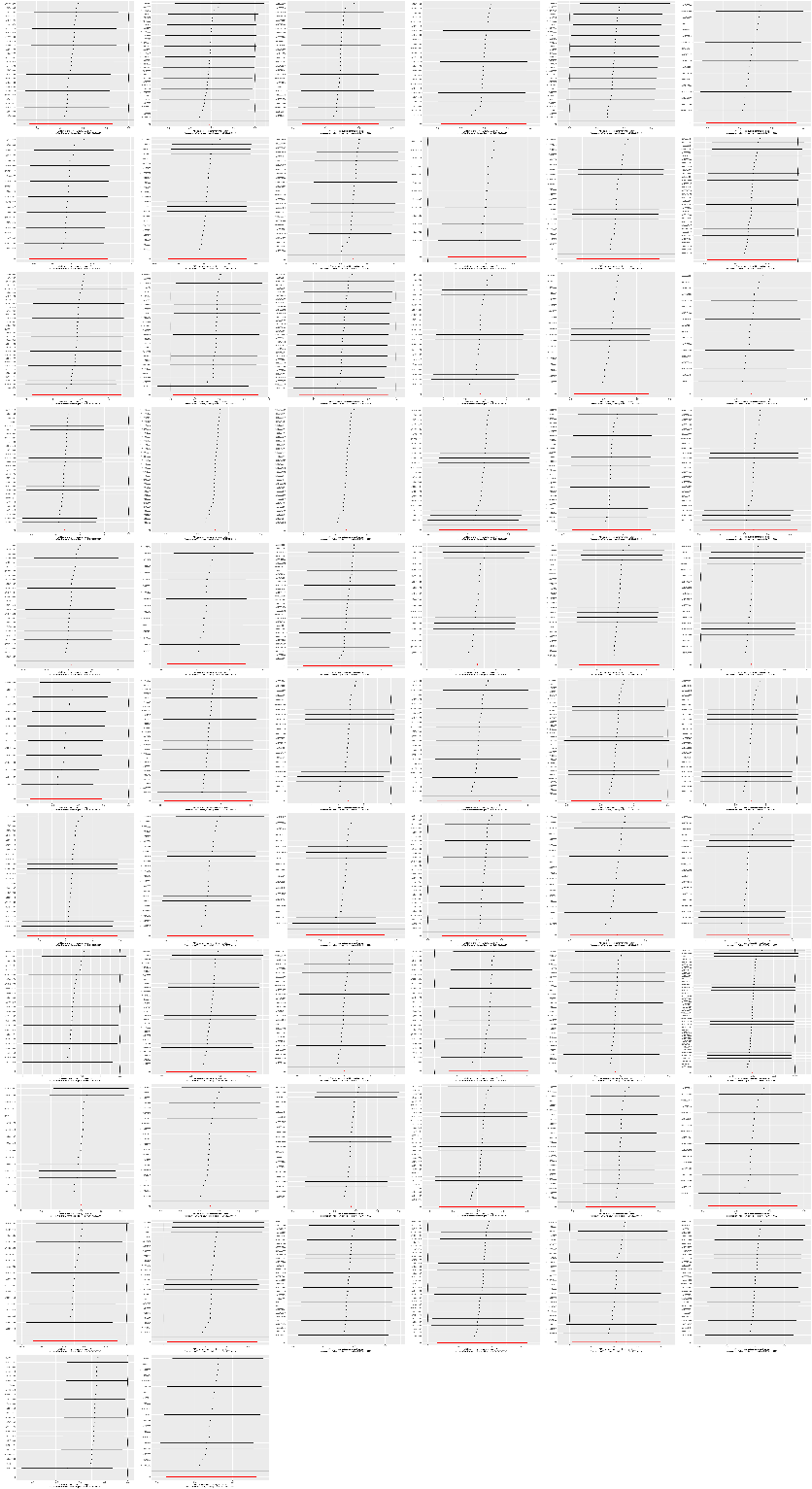


**Figure S1.** Sensitivity analyses plot


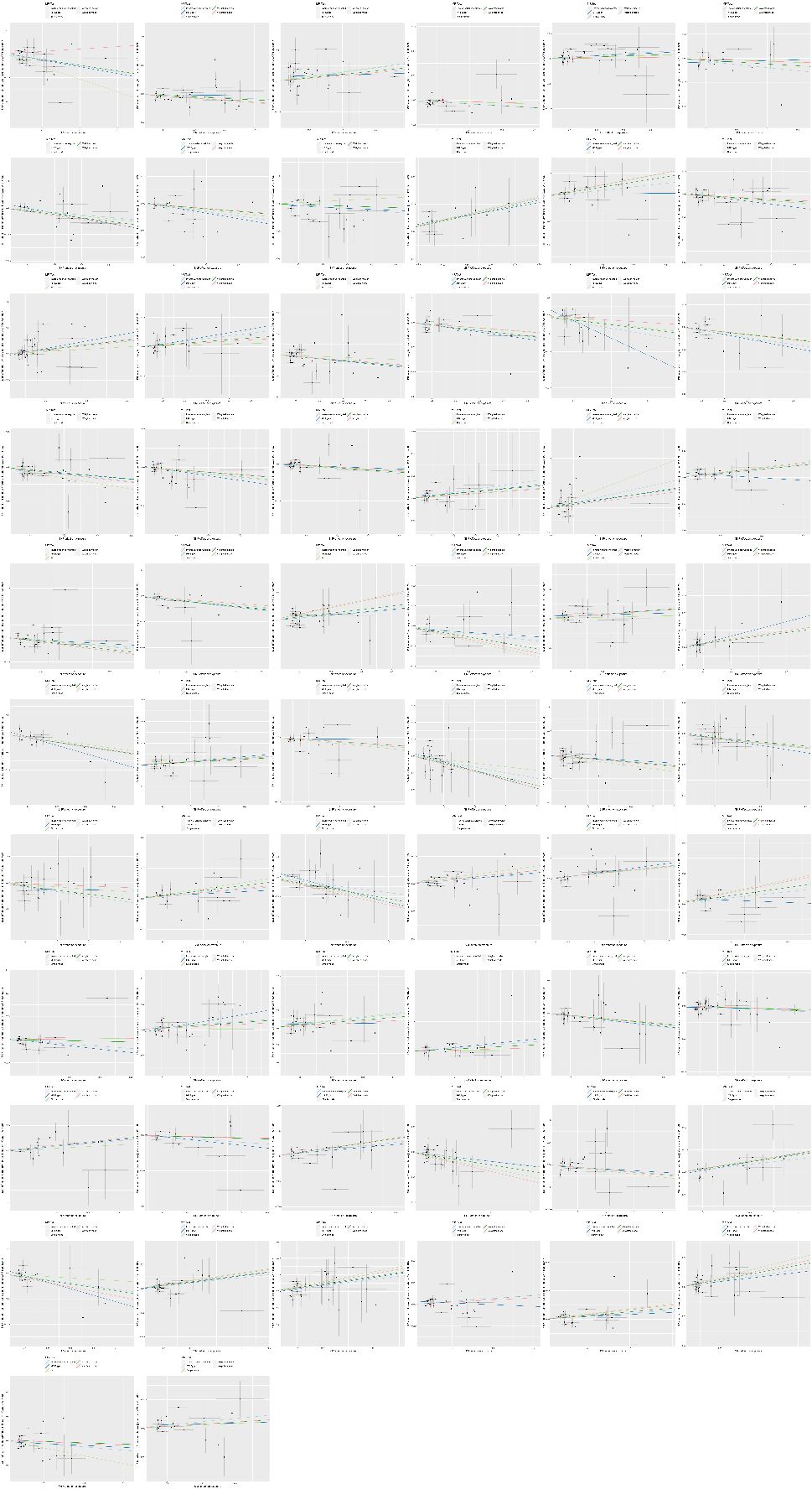


**Figure S2.** MR scatter plot


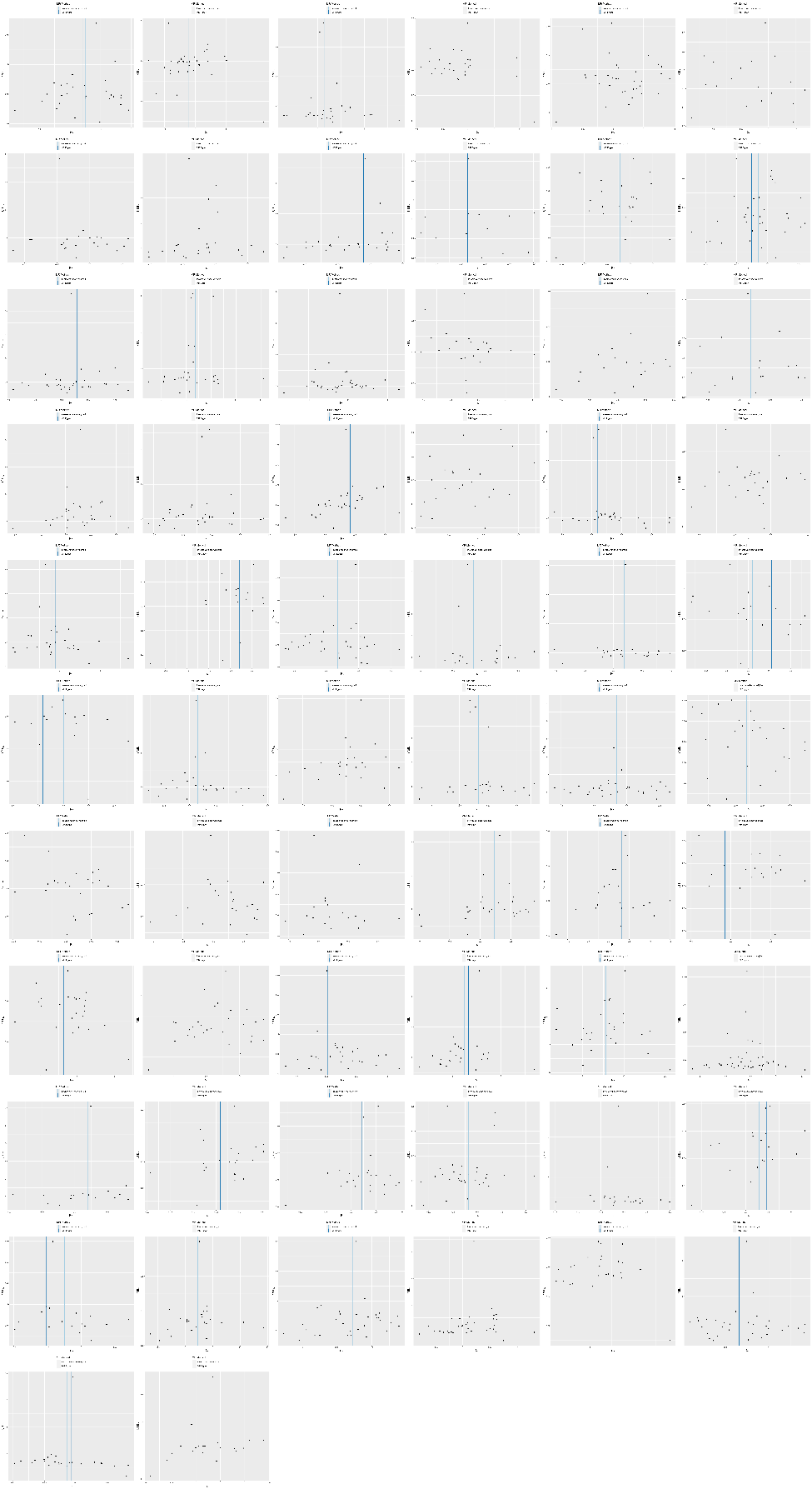


**Figure S3.** MR funnel plot


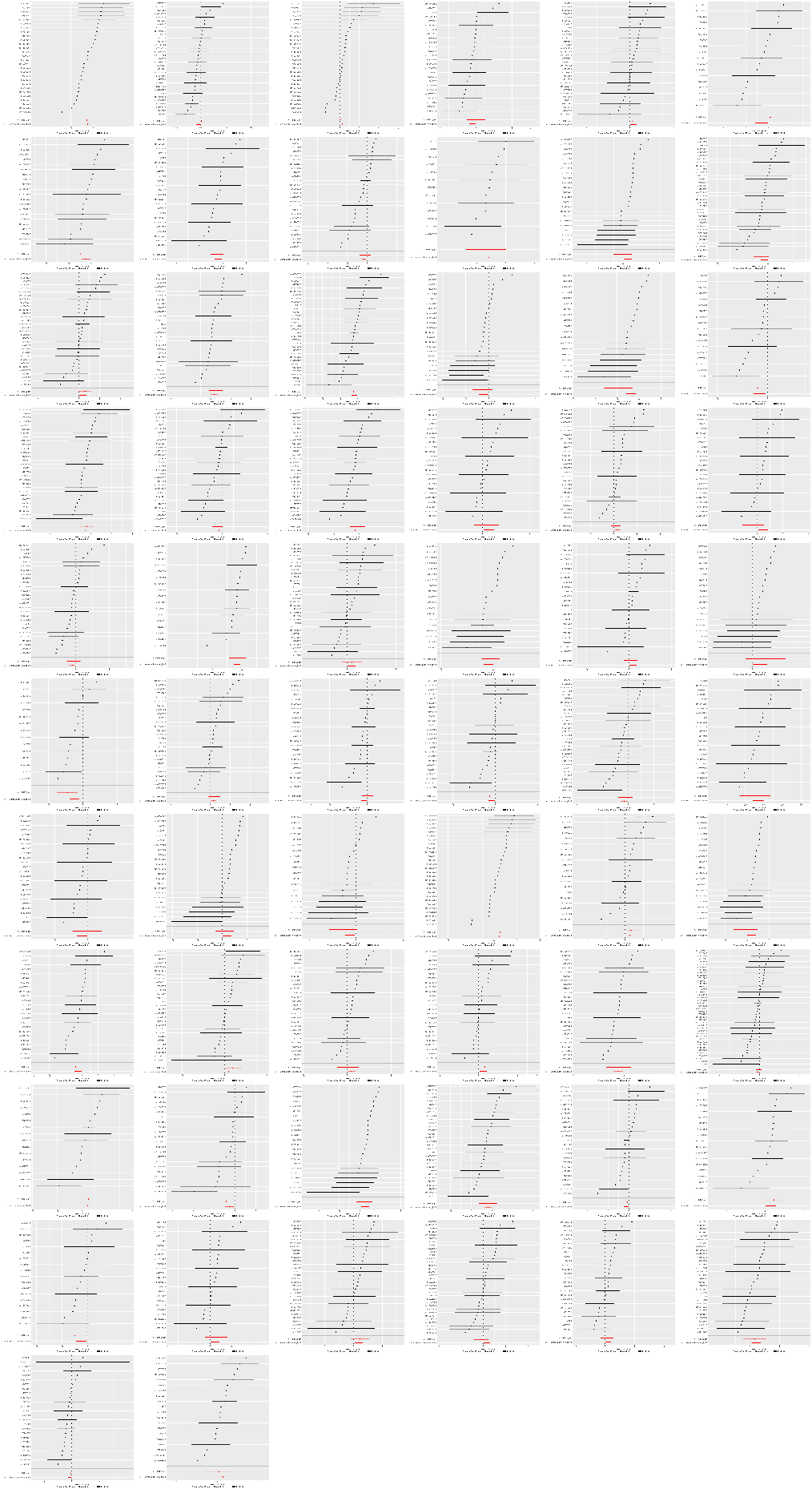


**Figure S4.** Forest plot
